# Can I get a witness?

**DOI:** 10.1186/s13054-014-0581-2

**Published:** 2014-10-28

**Authors:** Olufunmilayo Falade, Michael R Pinsky

**Affiliations:** The Clinical Research, Investigation, and Systems Modeling of Acute Illness (CRISMA) Center, University of Pittsburgh, Pittsburgh, PA 15261 USA; Department of Critical Care Medicine, University of Pittsburgh, Pittsburgh, PA 15261 USA

## Expanded abstract

### Citation

Jabre P, Belpomme V, Azoulay E, Jacob L, Bertrand L, Lapostolle F, Tazarourte K, Bouilleau G, Pinaud V, Broche C, Normand D, Baubet T, Ricard-Hibon A, Istria J, Beltramini A, Alheritiere A, Assez N, Nace L, Vivien B, Turi L, Launay S, Desmaizieres M, Borron SW, Vicaut E, Adnet F: Family presence during cardiopulmonary resuscitation. *N Engl J Med* 2013, 368:1008–1018.

### Background

The effect of family presence during cardiopulmonary resuscitation (CPR) on family members and the medical team remains controversial.

### Methods

The authors enrolled 570 relatives of active cardiac arrest patients receiving CPR by 15 pre-hospital emergency medical service units. The units were randomly assigned either to systematically offer the family member the opportunity to observe CPR (intervention group) or to follow standard practice regarding family presence (control group).

*Objective:* The primary end point was the proportion of relatives with post-traumatic stress disorder (PTSD)-related symptoms at 90 days. Secondary end points included the presence of anxiety and depression symptoms and the effect of family presence on medical efforts at resuscitation, the well being of the healthcare team, and the occurrence of medicolegal claims.

*Design:* Prospective cluster-randomized controlled trial.

*Setting:* Emergency medical service units were deployed to areas of the city across all socioeconomic groups in France from November 2009 to October 2011.

*Subjects:* Adult family members of adult patients in cardiac arrest occurring at home. Only one first-degree relative per patient was evaluated. The relative was chosen in accordance with the legislation on hospitalization at the request of a third party in the following order of preference: spouse, parent, offspring, sibling. Exclusion criteria were communication barriers with the relative and cardiac arrest cases in which resuscitation was not attempted.

*Intervention:* For emergency medical service units assigned to the intervention, a medical team member systematically asked family members whether they wished to be present during the resuscitation. A communication guide helped introduce the relative to the resuscitation scene and, when required, to help with the announcement of the death.

### Results

In the intervention group, 211 of 266 relatives (79%) witnessed CPR, compared with 131 of 304 relatives (43%) in the control group. In the intention-to-treat analysis, the frequency of PTSD-related symptoms was significantly higher in the control group than in the intervention group (adjusted odds ratio, 1.7; 95% confidence interval (CI), 1.2 to 2.5; *P* =0.004) and among family members who did not witness CPR than among those who did (adjusted odds ratio, 1.6; 95% CI, 1.1 to 2.5; *P* =0.02). Relatives who did not witness CPR had symptoms of anxiety and depression more frequently than those who did witness CPR. Family-witnessed CPR did not affect resuscitation characteristics, patient survival, the level of emotional stress in the medical team, and did not result in medicolegal claims.

### Conclusions

Family presence during CPR was associated with positive results on psychological variables of family members and did not interfere with medical efforts, cause increased stress in the healthcare team or result in medicolegal conflicts.

## Commentary

In the United States alone there are 450,000 cases of cardiopulmonary arrest annually. Eighty percent of these events occur at home with about 90% of the population dying and >50% of those that survive left with permanent brain damage [1]. In-hospital arrests have slightly better survival rates [2]. The desire to have family present during cardiopulmonary resuscitation (CPR) originates from 1987 when a family member insisted on being present during CPR [3]. There have since been several surveys designed to determine healthcare professionals’ preferences. Members of the healthcare team expressed reservations listing anxiety, family serving as barriers to provision of effective care and concerns that the family will suffer psychological ill effects. There has been increasing interest in the effect of family presence during CPR on both the family member and healthcare professionals with suggestions that it could be beneficial to the families as a grieving tool. In the pediatric world, family presence during CPR is the norm. However, resuscitation in pediatric patients differs from those in adults as survival rates are higher among children and the healthcare team is often prepared with the skills required to manage both the sick child and the anxious parent. Resuscitation guidelines advocate for the presence of families, including the European Resuscitation Council Guidelines for resuscitation and the American Heart Association guidelines for CPR and emergency cardiovascular care [4,5]. Thus, Jabre and colleagues conducted the first randomized trial of its kind to understand the effects family presence during CPR has on both the family and the healthcare team.

This study was a well-designed prospective cluster randomized controlled trial and took place in France over 2 years. The primary end point was the presence of post-traumatic stress disorder (PTSD) in the relatives that witnessed CPR at 90 days. The secondary end point was the presence of anxiety and depression in the relatives of the decedents, the well-being of the healthcare team and occurrence of medicolegal claims. The intention-to-treat analysis showed that family members in the control group were more likely to exhibit symptoms of PTSD than those in the intervention group with an odds ratio (OR) of 1.7 (confidence interval (CI) 1.2 to 2.5). Family members who did not witness CPR were more likely to exhibit symptoms of PTSD compared with those that did with an OR of 1.6 (CI 1.1 to 2.5). The study also concluded that family presence did not interfere with medical resuscitation efforts, increase stress among members of the medical team, or result in additional medicolegal conflicts.

This study had several strengths. First, the inclusion and exclusion criteria were simple and could be rapidly applied in the field. Second, the sample size was adequately powered to observe the desired effect. Third, psychological effects of witnessing CPR or not was assessed using well-evaluated monitoring tools. The study had one significant weakness and a few minor ones. Importantly, there was significant crossover between the intervention and the control group with about 43% of the control group opting to witness CPR. They are listed as getting standard of care but it is unclear what standard care entails and more importantly how that differs from the intervention, especially as the controls were given communication guides too. This raised concern as to what the intervention really was: was it the presence of family during CPR or the presence of a communication guide for the families or both? Other limitations include that the observed variables are subject to cultural differences that may not allow direct extrapolation to North American populations. For example, does being a predominantly Catholic population in a largely paternalistic healthcare system inform patients/relatives responses to the healthcare team and end of life decisions? Randomization of the emergency medical service teams *ab initio* also allowed for the potential to introduce selection bias. Regardless, psychological benefits persisted for family members offered the possibility to witness the CPR of a relative in a follow-up study performed 1 year after the event [6].

This study is an important addition to the evidence in support of family member presence during CPR. In tandem with release of the results of this study, the *New England Journal of Medicine* ran a clinical vignette about a middle aged woman that required CPR and opened voting polls to determine the opinions of their readership. Despite the results of this article, an overwhelming number of respondents (69%) maintained that they do not think family should be allowed to be present when CPR is being performed on their relatives. Interestingly, France had the majority of respondents in support of family presence during CPR. It is unclear if this is a reflection of the opinion of members of the research group or the general population. This unfortunately suggests that healthcare teams will continue to opt for exempting family members from this sentinel event in the lives of their relatives. This approach may stem from the wish to protect the family members from viewing potentially traumatic therapy that often is unsuccessful. However, cultural differences in physician and population preferences across geographical regions limit broad extrapolation of these results.

## Recommendation

The overwhelming number of patients that do not survive cardiac arrest or survive with significant neurologic sequelae makes cardiac arrest an end of life event requiring interventions designed to facilitate the grieving process. We recommend that in the appropriate circumstance, family should be offered the opportunity to be present during CPR. Specific protocols should be instituted to manage this event and a ‘communication guide’ as used in the trial should be with the family until CPR is terminated.

## Abbreviations

CI: Confidence interval
CPR: Cardiopulmonary resuscitation
OR: Odds ratio
PTSD: Post-traumatic stress disorder
